# Cortical activation in elderly patients with Alzheimer’s disease dementia during working memory tasks: a multichannel fNIRS study

**DOI:** 10.3389/fnagi.2024.1433551

**Published:** 2024-09-25

**Authors:** Nairong Ruan, Xingxing Li, Ting Xu, Zheng Zhao, Xi Mei, Chengying Zheng

**Affiliations:** ^1^Department of Psychiatry, Affiliated Kangning Hospital of Ningbo University, Ningbo, China; ^2^Department of Psychiatry, Ningbo Kangning Hospital, Ningbo, China

**Keywords:** cortical activation, functional connectivity, working memory, Alzheimer’s disease, functional near-infrared spectroscopy

## Abstract

**Objective:**

This study aimed to investigate cortical activation and functional connectivity in the cortex during working memory (WM) tasks in patients with Alzheimer’s disease (AD) using functional near-infrared spectroscopy (fNIRS).

**Methods:**

A total of 17 older adults with AD and 17 cognitively normal (CN) participants were recruited. fNIRS was utilized to monitor oxygenated hemoglobin (HbO) concentrations in the frontotemporal lobe, while participants performed WM tasks to examine WM impairments in subjects with AD. Student’s t-test for continuous variables and the chi-square test for categorical variables were used to compare the clinical and HbO variables between the AD and CN groups. Functional connectivity was analyzed using Pearson’s correlation coefficient between the time series of each channel-to-channel pair.

**Results:**

The changes in HbO concentrations and cortical activations during the WM task showed that the HbO concentration curve of the CN group was higher than that of the AD group during the encoding and maintenance phases of the WM task. Although in the brain region scale, there were no significant differences in average HbO concentrations between the two groups, many channels located in the frontal and temporal lobes showed significant differences (*p* < 0.05) in the average HbO (channels 7 and 32) and slope HbO values (channels 7, 8, 9, 23, 30, 34, and 38) during the WM task. The average functional connectivity of the AD group was significantly lower than that of the CN group (*p* < 0.05). The functional connectivity was stronger in the frontopolar (FP) region than in other areas in both groups.

**Conclusion:**

This study revealed there were significant differences in HbO concentration in older adult patients with AD compared to CN during the WM task. The characteristics of HbO measured by the fNIRS technique can be valuable for distinguishing between AD and CN in older adults.

## 1 Introduction

AD is a prevalent neurodegenerative disorder and the primary cause of dementia (Scheltens et al., 2021). Its main symptoms include cognitive impairment and difficulties with daily tasks (Benmelouka et al., 2022). Cognitive impairment can affect functions such as visuospatial function, memory, attention, executive functioning, and learning (Dhakal and Bobrin, 2024). Memory loss is noticeable even in mild cognitive impairment (MCI), which is considered an early stage of AD (Gold and Budson, 2008; Gagliardi and Vannini, 2021). Severe cognitive decline significantly impacts the ability of older adults to live independently. Due to its high incidence and prevalence rates, AD is quickly becoming one of the most expensive and burdensome diseases worldwide (Alzheimers Dement, 2023).

*β*-amyloid (Aβ) plaques and neurofibrillary tangles are related to neural activation (Richter et al., 2020). At the early stage of AD or MCI, memory impairments and cognition deficits are likely caused by impaired neuronal activity rather than by cell death (Igarashi, 2023). Even in other types of dementia, brain structural changes were usually accompanied by cortical activation decline (Musaeus et al., 2021). Previous studies found that many stimulation programs, including repetitive transcranial magnetic stimulation (rTMS) and sensory stimuli, May slow down cognitive and functional decline by elevating the cortical activations (Koch et al., 2022; Chou et al., 2020; Steffener et al., 2021).

Apart from the identification of new blood-based and imaging biomarkers, the diagnosis of AD could potentially incorporate neuropsychological biomarkers, such as abnormal WM characteristics. WM is a cognitive system responsible for manipulating information storage and processing through encoding, maintenance, and retrieval (Kim, 2019). Furthermore, it is the foundation for advanced cognitive functions, such as learning, reasoning, and decision-making (Müller et al., 2021). WM is impaired in older adults across the AD continuum, including those with MCI and early AD, highlighting these neurocognitive impairments (Kirova et al., 2015; Germano and Kinsella, 2005; Huntley and Howard, 2010).

Electroencephalogram (EEG) studies on WM have been conducted in different individuals, including those with cognitive disorders (Hsieh and Ranganath, 2014; Ruimin et al., 2017). These results suggest that changes in EEG power or band can reflect the brain function in a high WM load (Wei and Zhou, 2020). Neuroimaging studies, such as functional magnetic resonance imaging (fMRI), on memory impairment in AD, have indicated that function in brain regions critical to episodic memory is altered in AD (Rémy et al., 2005). These functional changes May closely correlate with the progressive structural changes observed in the hippocampal region (de Toledo-Morrell et al., 2000). Previous fMRI studies indicated that, compared to healthy older adults, participants with MCI showed decreased activation in the bilateral prefrontal and temporoparietal cortices during the WM tasks (Lou et al., 2015; MACHULDA et al., 2009). Positron emission tomography (PET) studies also showed that Aβ deposits were associated with neural cognitive function during WM tasks (Li et al., 2023).

Although the abovementioned neuroimaging techniques had many advantages in the study of WM in AD patients, a new type of neuroimaging technology is gradually being used. In recent years, brain activation of patients with cognitive impairment can be explored using a new non-invasive detection of fNIRS (Niu et al., 2013; Yang et al., 2019). Advantages of fNIRS over EEG and fMRI included better portability, comfort, less noise, low cost, high temporal resolution, and being insensitive to motion (Hu et al., 2020; Pinti et al., 2020). It was suitable for the elderly and even superior to amyloid PET and fMRI in some aspects (Kim and Yon, 2022). Moreover, fNIRS is capable of providing comparable functional connectivity measures to fMRI (Duan et al., 2012). fNIRS can reveal resting HbO concentrations and task-related changes in HbO concentrations in patients with MCI and dementia (Yeung and Chan, 2020). fNIRS can also comprehensively investigate functional connectivity in the prefrontal cortex during a WM task (Yu and Lim, 2020).

This study aims to use the fNIRS to investigate the prefrontal, temporal, and partial parietal cortex activity in patients with AD during WM tasks and to explore the strength of functional connectivity between different brain regions during WM tasks.

## 2 Methods

### 2.1 Participants

This study enrolled 34 participants, consisting of 17 patients with AD and 17 CN subjects according to the sample size calculation and previous fNIRS studies on AD (Wang and Ji, 2020; Cicalese et al., 2020; Keles et al., 2022). Participants were recruited from the Geriatric Psychiatric Center of the Affiliated Kangning Hospital of Ningbo University between January and December 2023. The diagnosis of AD was based on the criteria outlined in the Diagnostic and Statistical Manual of Mental Disorders, Fifth Edition. All participants met the following criteria: (1) diagnosis confirmed by two research psychiatrists, (2) provided informed consent, and (3) had a disease course >6 months. Through brief face-to-face interviews, a healthy control group of cognitively unimpaired participants was confirmed to exhibit no cognitive decline. All procedures were in accordance with the ethical standards of the ethics committee of Affiliated Kangning Hospital of Ningbo University. The patients/participants [legal guardian/next of kin] provided written informed consent to participate in this study.

### 2.2 Neurocognitive assessments

The cognitive levels of the participants were assessed using the Mini-Mental State Examination (MMSE) and Alzheimer’s Disease Assessment Scale-cognitive subscale (ADAScog) (Jia et al., 2021; Cogo-Moreira et al., 2023). MMSE scores range from 0 to 30, with higher scores indicating better cognitive function. MMSE scores <24 ~ 25 suggested dementia (Folstein et al., 1975; Cockrell and Folstein, 1988). The ADAScog cutoff value for distinguishing mild and moderate dementia is 22. Higher scores of ADAScog suggested poorer cognitive function (Rosen et al., 1984).

### 2.3 fNIRS detection and working memory protocol

**Figure 1:**
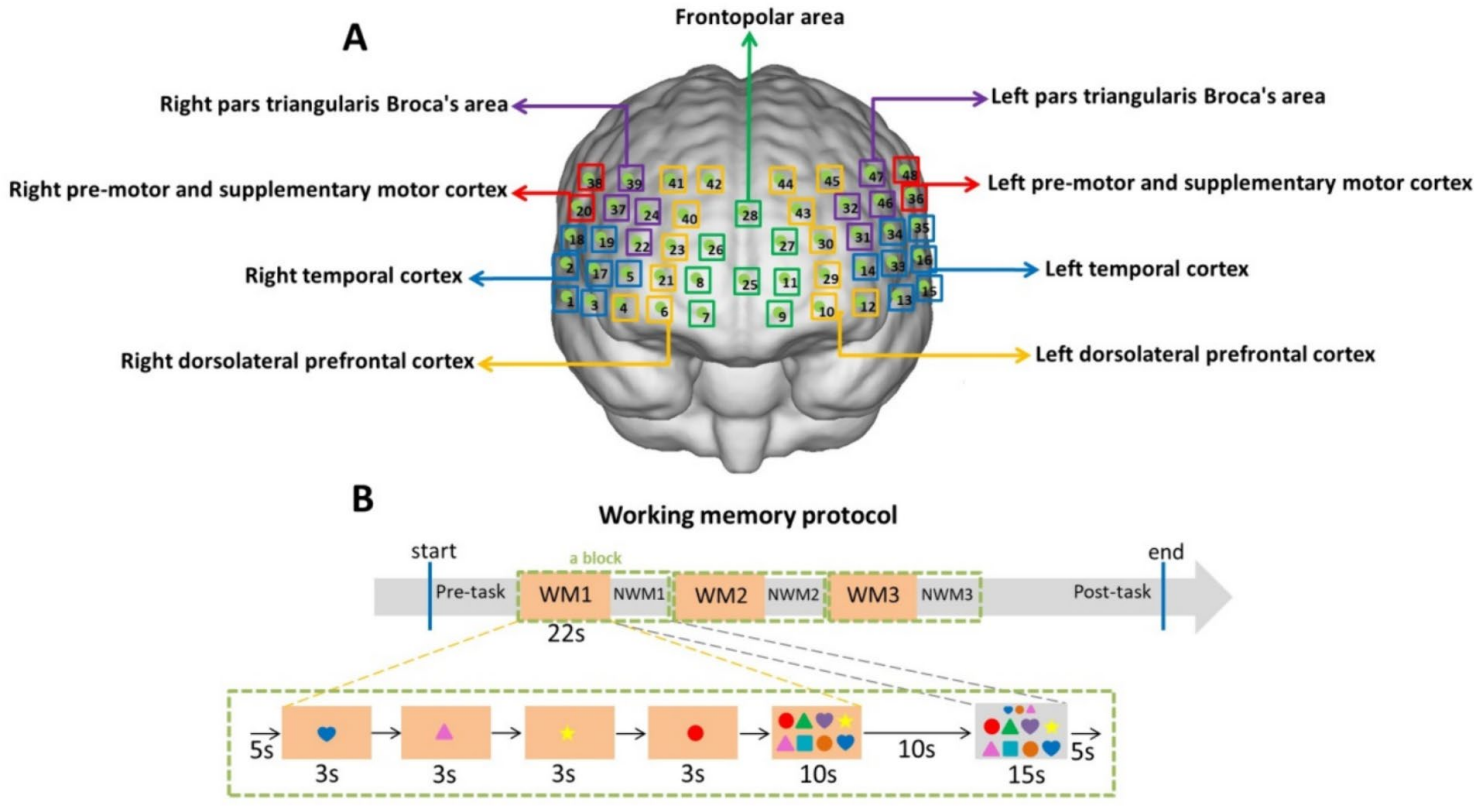
Location of detection **(A)** and working memory protocol **(B)**. **(A)** Green boxes represent the frontopolar area (channels 7, 8, 9, 11, 25, 26, 27, and 28); purple boxes represent the left and right pars triangularis Broca’s area (22, 24, 37, 39 on the right and 31, 32, 46, 47 on the left); red boxes represent the left and right pre-motor and supplementary motor cortex (20, 38 on the right and 36, 48 on the left); blue boxes represent the left and right temporal cortex (1, 2, 3, 5, 17, 18, 19 on the right and13, 14, 15, 16, 33, 34, 35 on the left); yellow boxes represent the left and right dorsolateral prefrontal cortex (4, 6, 21, 23, 40, 41, 42 on the right and 10, 12, 29, 30, 43, 44, 45 on the left). **(B)** The working memory task structures comprised three blocks. A block (the green dotted box) consisted of 5 s resting time, 12 s encoding time (4 pictures × 3 s), 10 s retrieval time, 10 s resting time, 15 s non-working memory (NWM) time, and 5 s resting time. The baseline was the first 5 s resting time of the block.

Figure 1 illustrates the location of the detection (Figure 1A) and the protocols for the WM task (Figure 1B) used in this study. Our previous study measured HbO concentrations using a multichannel functional near-infrared brain imaging device (NirScan-6000A; Danyang Huichuang Medical Equipment Co., Ltd., China) (Liu et al., 2022). The sampling frequency was 11 Hz; 730 and 850 nm were the major wavelengths, and 808 nm was used as the isotopic wavelength for correction, as previously described (Liu et al., 2022). We used the FPz channel (10/20 International System) as the center of the middle probe and positioned 31 SD probes (comprising 15 sources and 16 detectors) with a fixed interprobe distance (3 cm) to cover the bilateral prefrontal and temporal cortices of all the participants. In this study, 48 NIRS channels were established. The brain regions and corresponding channels are shown in Figure 1A. Nine regions of interest were delineated according to the Brodmann map and fNIRS system settings (Bruner, 2022; Rorden and Brett, 2000; Wu et al., 2022). As shown in Figure 1A, the FP area contained channels 7, 8, 9, 11, 25, 26, 27, and 28; the right pars triangularis Broca’s area contained channels 22, 24, 37, and 39; the right pre-motor and supplementary motor cortex contained channels 20 and 38; the right temporal cortex (RTC) contained channels 1, 2, 3, 5, 17, 18, and 19; the right dorsolateral prefrontal cortex (RDLPFC) contained channels 4, 6, 21, 23, 40, 41, and 42; the left pars triangularis Broca’s area contained channels 31, 32, 46, and 47; the left pre-motor and supplementary motor cortex contained channels 36 and 48; the left temporal cortex (LTC) contained channels 13, 14, 15, 16, 33, 34, and 35; the left dorsolateral prefrontal cortex (LDLPFC) contained channels 10, 12, 29, 30, 43, 44, and 45.

As described previously, the WM task was performed with participants seated comfortably in a quiet room and completing the task on a tablet (Liu et al., 2024). The subject will be required to operate on Microsoft Surface. As shown in Figure 1B, the WM task structures comprised three blocks (the green dotted box). A block consisted of two periods: the WM period and the non-working memory (NWM) period. During the WM period, four color shapes (each displayed for 3 s) will appear, and the subject is required to memorize their color, shape, and order of appearance (the encoding and maintenance phases). Subsequently, the subject will be asked to click on the integrated image in the order they remember, within 10 s (the retrieval phase). During the 15-s NWM period, the subject will be asked to click on a similar integrated image according to a shapes array shown above. The time duration of the two periods is shown in Figure 1B. Although the baseline was 5 s, there was a long resting time before the baseline. We chose 5 s as the baseline. Three blocks were set in the WM protocol to avoid the inaccuracies caused by participants’ fatigue. The WM and NWM switching can increase the cognitive load (Yanagisawa et al., 2016; Naoko Narita et al., 2012).

### 2.4 fNIRS data preprocessing

The NirSpark software package (V1.7.5, Hui Chuang, China) was used to analyze the fNIRS data. The data were preprocessed, and motion artifacts were corrected using the moving SD and cubic spline interpolation methods. A bandpass filter with a 0.01–0.20 Hz cutoff frequency was used to remove physiological noise. The modified Beer–Lambert law was used to calculate the relative changes in hemoglobin concentrations in the HbO and HbR. Instead of HbR, we used HbO as our primary indicator in the following analysis due to its high signal-to-noise ratio.

The mean HbO concentration was calculated using 22-s task periods of the WM phases. The slope of the HbO concentration in the WM indicated the increase in HbO within 5 s of the start of the WM task. Linear fitting was applied to the data of the two baselines. Subsequently, the mean waveform of HbO changes in each channel was derived from the waveforms of individuals in all 48 channels for all participants in the two groups. The topographic maps of HbO concentration were drawn using the NirSpark software version 1.7.5 (Danyang Huichuang Medical Equipment Co., Ltd., China). The topographic maps were created using the HbO concentration features (e.g., mean HbO concentration change during the activation period and the slope of HbO concentration change during the activation period) detected from individual probes.

### 2.5 Statistical analysis

The data are presented as mean ± standard deviation (SD). Demographic and clinical variables were compared between the two groups using Student’s t-test. A t-test was also used to compare HbO changes between patients and CN participants to evaluate brain activation between the two groups. Functional connectivity was analyzed by the NirSpark software package (V1.7.5, Hui Chuang, China) using Pearson’s correlation coefficient between the time series of each channel-to-channel pair. The statistical results were corrected for multiple comparisons across channels using the false discovery rate. Statistical significance was set at a *p*-value of <0.05. All analyses were performed using the Statistical Package for the Social Sciences software (SPSS version 19.0; IBM Corp., Armonk, NY, United States).

## 3 Results

### 3.1 Clinical characteristics

**Figure 2:**
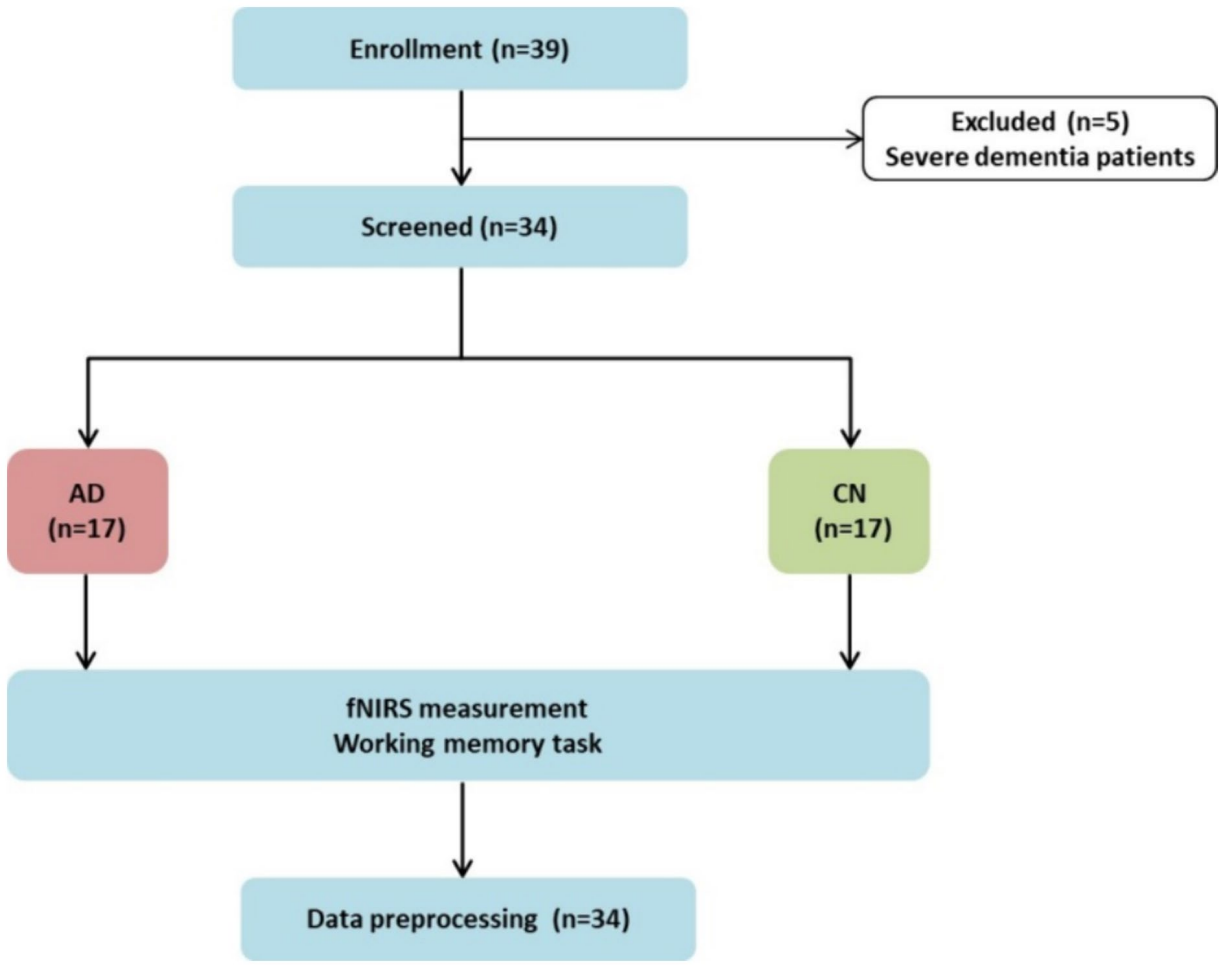
Flowchart of the study.

Patients were recruited for this study from November 2023 to April 2024 at the Geriatric Psychiatric Department of the Affiliated Kangning Hospital of Ningbo University. Figure 2 illustrates a flowchart depicting the participants and protocol. Five patients were unable to complete the WM task due to severe dementia. A total of 34 participants were included in this study. The demographic characteristics of the participants are presented in Table 1. The demographic variables in the AD and CN groups showed no differences in mean age, sex, or educational level (*p* > 0.05). With regard to the neuropsychiatric scales scores, the AD group exhibited significantly lower scores on MMSE (20.06 ± 2.90, *t* = −11.966, *p* < 0.001) and higher scores on the ADAScog (30.27 ± 13.18, *t* = 7.230, *p* < 0.001) than the CN group.

**Table 1:** Demographic and clinical data.

Variables	AD group	CN group	t/Chi-square value	*p*-value
Age (year)	76.24 ± 6.68	72.71 ± 5.38	1.697	0.099
Sex (m/f)	6/11	8/9	1.059	0.303
Education (year)	5.29 ± 3.09	6.18 ± 2.38	−0.932	0.359
MMSE score	20.06 ± 2.90	29.06 ± 1.09	−11.966	0.000
ADAScog score	30.27 ± 13.18	6.68 ± 2.69	7.230	0.000

The data were presented as mean ± standard deviation (SD); AD, Alzheimer’s disease; CN, cognitive normal; MMSE, Mini-Mental State Examination; ADAScog, Alzheimer’s Disease Assessment Scale-cognitive subscale.

### 3.2 Changes in HbO concentrations during working memory tasks

**Figure 3:**
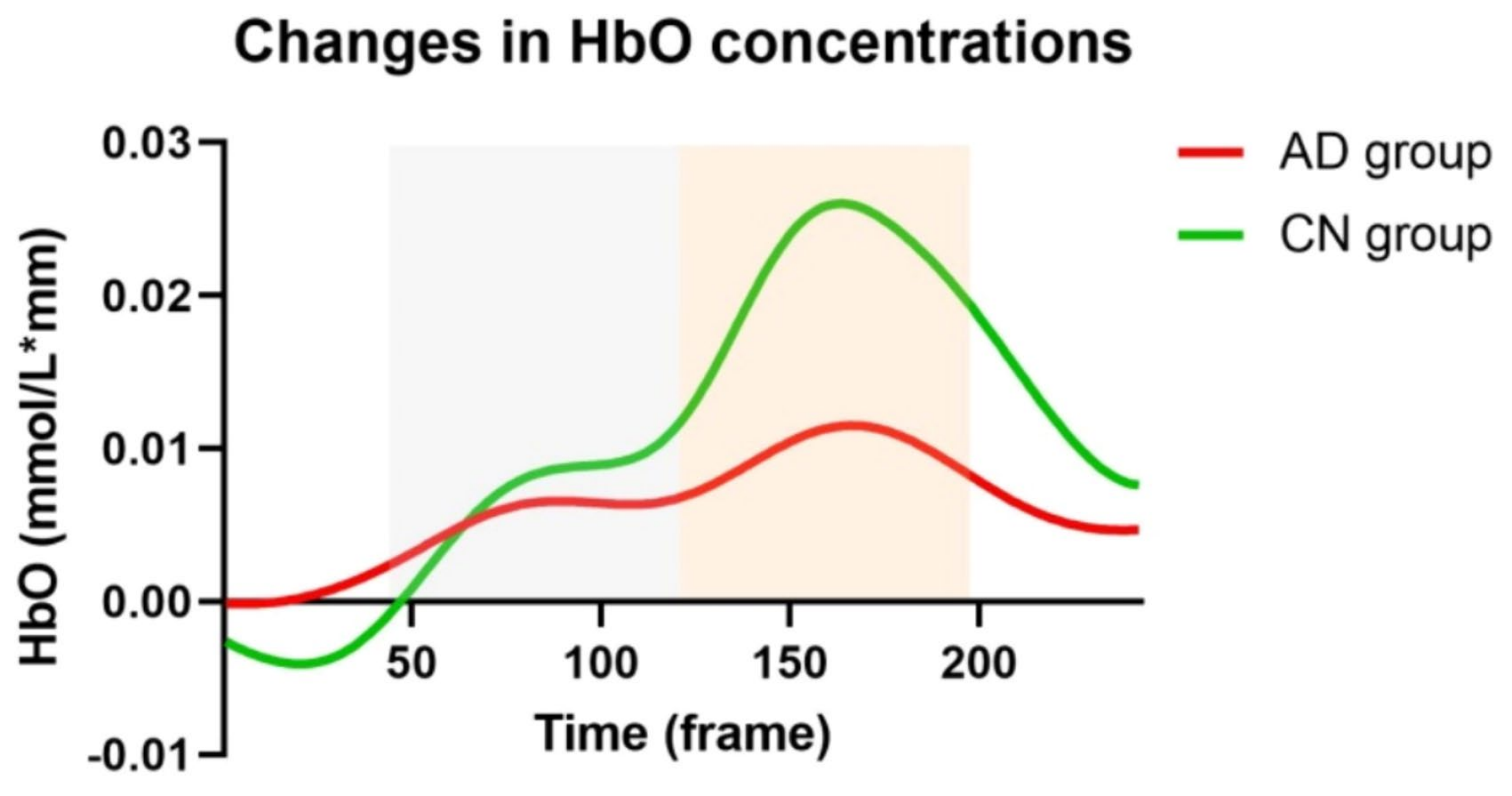
Changes in HbO concentrations during working memory tasks in AD and CN groups. Red and green lines represent AD and CN groups, respectively. Gray and orange dashed areas represent the encoding and retrieval phases during working memory tasks.

Figure 3 illustrates the changes in HbO concentrations in the AD and CN groups during the WM task. Prior to the commencement of the WM tasks, HbO concentrations were higher in the AD group than in the CN group. This value represents a relative change of the task-state HbO concentration relative to the resting-state HbO concentration. This suggested a lower activation before the WM task in the CN group. During the WM tasks, there were two rising phases of the HbO concentration, representing the encoding and retrieval phases. The CN group demonstrated higher HbO concentrations than the AD group. Additionally, the time of responses in the two groups was nearly synchronized.

We performed the t-test for four variables, including mean HbO concentrations of the encoding phase and retrieval phase in AD and CN groups, respectively. There were significant differences in mean HbO concentrations of the encoding phase (*t* = 5.515, *p* < 0.001) and retrieval phase (*t* = 28.51, *p* < 0.001) between AD and CN groups. Meanwhile, there were significant differences in mean HbO concentrations between the encoding phase and retrieval phase both in AD (*t* = 21.12, *p* < 0.001) and CN (*t* = 27.90, *p* < 0.001) groups.

### 3.3 HbO concentrations in different brain regions

**Figure 4:**
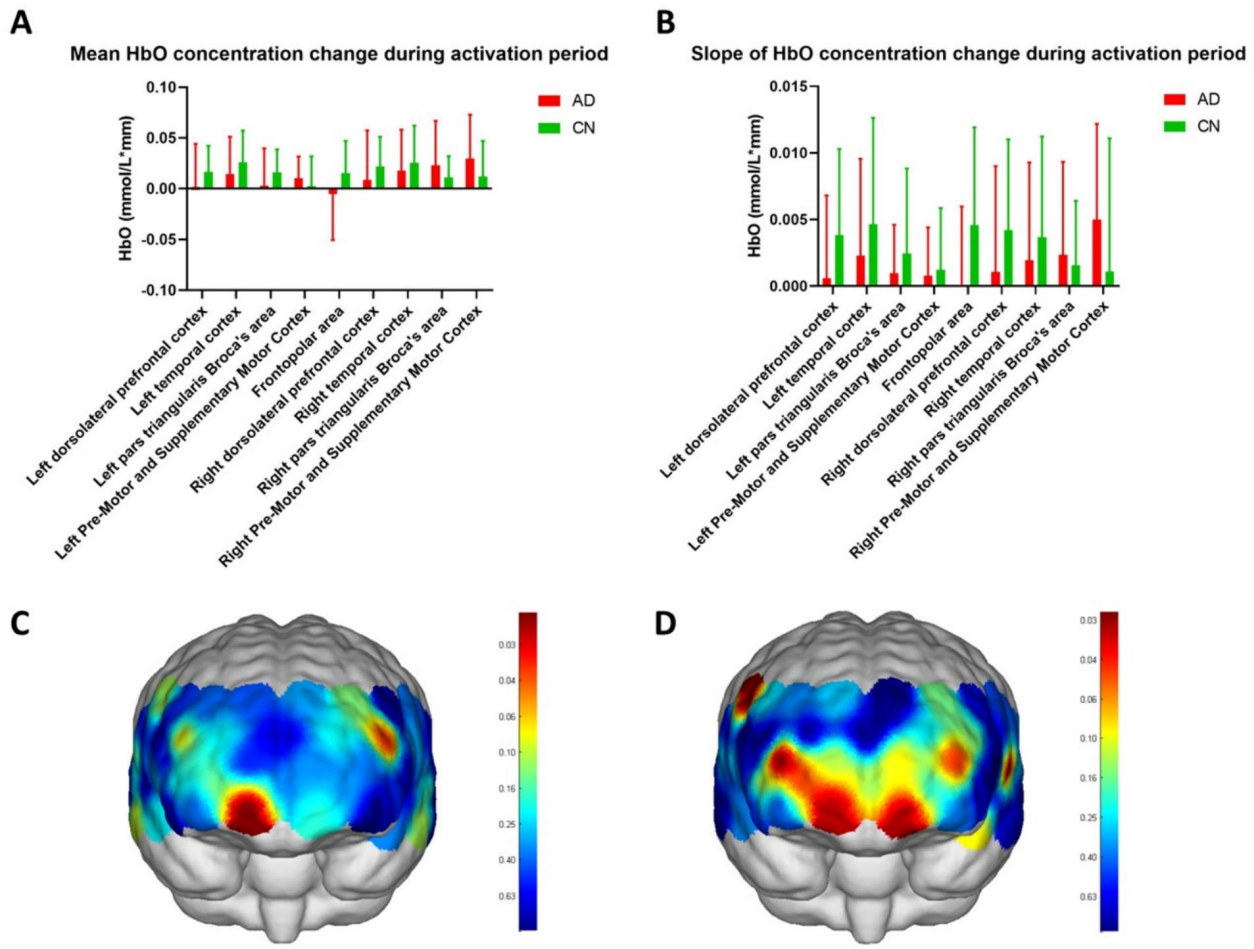
HbO concentrations of variable brain regions. **(A,B)** show the mean HbO concentration change during the activation period and slope of HbO concentration change during the activation period of nine regions in the frontal and temporal lobes, including the frontopolar area, left and right dorsolateral prefrontal cortex, left and right temporal cortex, left and right pars triangularis Broca’s area, and left and right pre-motor and supplementary motor cortex. **(C,D)** show the topographic maps of mean HbO concentration change during the activation period and slope of HbO concentration change during the activation period and show the significant difference in channels between the two groups in red color. The scale bars in **C,D** represent the *p*-value.

In Figure 4, the mean HbO concentration change during the activation period was calculated using task periods of the WM phases and represented the average HbO concentration during the WM task. The slope of the HbO concentration change during the activation period indicated the increase in HbO concentration within 5 s of the start of the WM task and reflected the ability to respond to the task. Figures 4A,B depict the HbO concentrations in different brain regions. Except for the right pars triangularis Broca’s area and the right pre-motor and supplementary motor cortices, both the average (Figure 4A) and slope (Figure 4B) values of the HbO concentration in WM tasks were higher in the CN group than the AD group. Although no significant difference was observed in the HbO concentrations across different brain regions between the two groups, the HbO concentrations across the channels showed significant differences (Figures 4C,D, Table 2, *p* < 0.05) in WM average (Table 2, mean HbO concentration change during activation period, channels 7 and 32) and slopes (Table 2, The slope of HbO concentration change during activation period, channels 7, 8, 9, 23, 30, 34, and 38) locating in frontal and temporal lobes. Figure 4C indicates that the location of channels 7 and 32 showed a significant difference in mean HbO concentration change during the activation period between the two groups. Channels 7 and 32 are located in the FP area and left pars triangularis Broca’s area, respectively. Figure 4D suggests that the location of channels 7, 8, 9, 23, 30, 34, and 38 showed a significant difference in the slope of HbO concentration change during the activation period between the two groups. Channels 7, 8, and 9 are located in the FP area. Channels 23 and 30 are located in the right and left dorsolateral prefrontal cortex (RDLPFC and LDLPFC), respectively. Channel 34 is located in the LTC. Channel 38 is located in the right pre-motor and supplementary motor cortex.

**Table 2:** Channels that showed significant differences during working memory tasks.

Channel numbers	AD group (*n* = 17)	CN group (*n* = 17)	*t*-value	*p*-value
Mean HbO concentration changes during the activation period				
S3-D3 (chan 7)	−0.025 ± 0.067	0.022 ± 0.038	−2.533	0.017
S10-D15 (chan 32)	−0.014 ± 0.065	0.031 ± 0.052	−2.218	0.034
Slope of HbO concentration changes during the activation period				
S3-D3 (chan 7)	−0.001 ± 0.007	0.007 ± 0.012	−2.305	0.028
S3-D8 (chan 8)	−0.001 ± 0.006	0.006 ± 0.011	−2.081	0.045
S4-D3 (chan 9)	−0.001 ± 0.006	0.006 ± 0.010	−2.329	0.026
S8-D8 (chan 23)	−0.001 ± 0.009	0.007 ± 0.011	−2.384	0.023
S10-D9 (chan 30)	−0.001 ± 0.008	0.004 ± 0.007	−2.083	0.045
S11-D10 (chan 34)	−0.000 ± 0.005	0.005 ± 0.007	−2.297	0.028
S12-D12 (chan 38)	0.006 ± 0.009	−0.000 ± 0.006	2.374	0.024

Data were presented as mean ± standard deviation (SD); AD, Alzheimer’s disease; CN, cognitive normal.

### 3.4 Functional connectivity during working memory tasks

**Figure 5:**
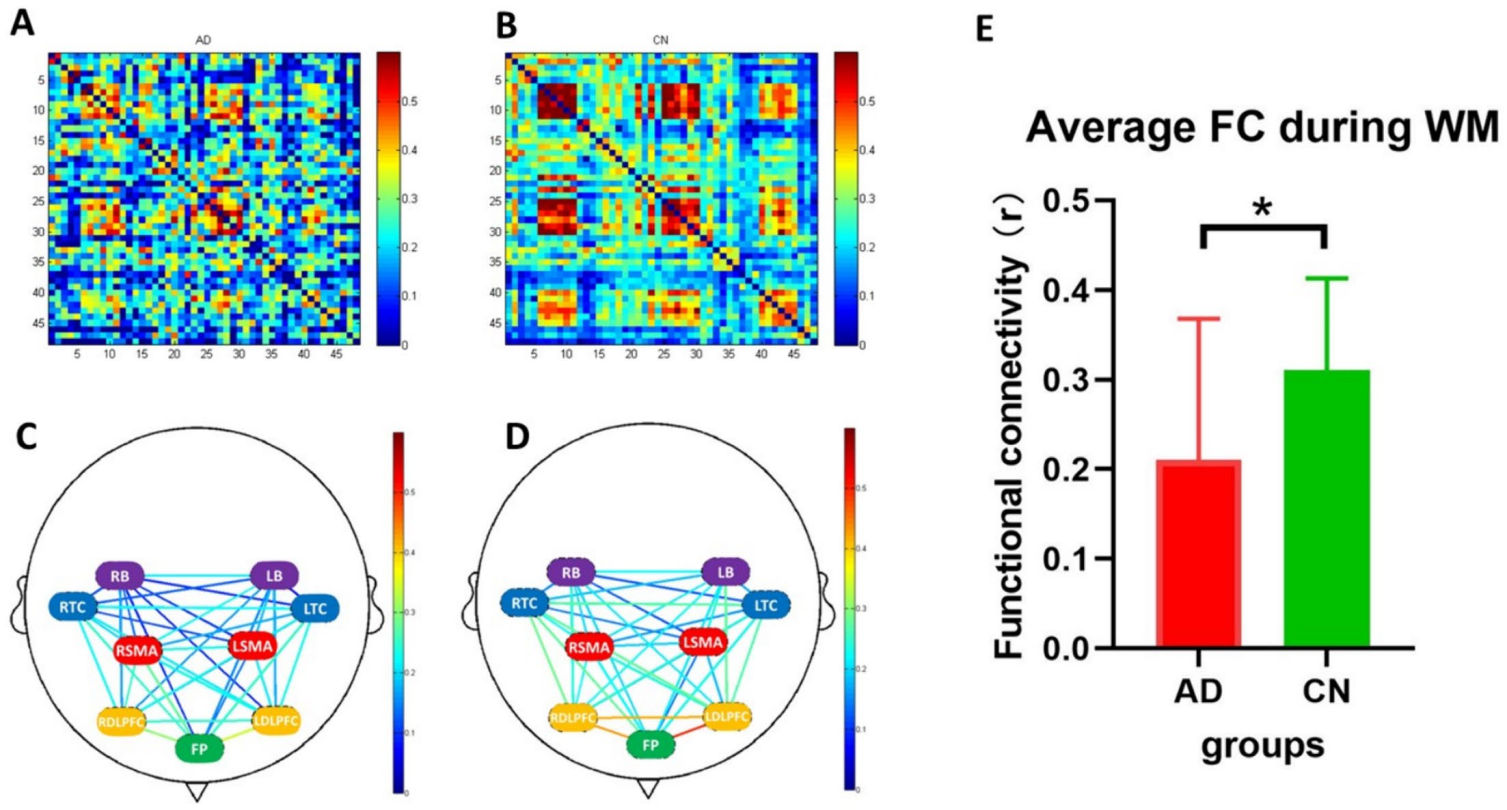
Functional connectivity during working memory tasks. **(A,B)** are heatmaps of 48 channels (the x and y axes represent the channel numbers) in AD and CN groups, and **(C,D)** are connection diagrams of the two groups. **(E)** Represents the average functional connectivity, which showed a significant difference between AD (red bar) and CN (green bar) groups (*p* < 0.05). FC, functional connectivity; WM, working memory; *, < 0.05. In C and D, green represents the frontopolar (FP) area; purple represents the left and right pars triangularis Broca’s area (LB and RB); red represents the left and right pre-motor and supplementary motor cortex (LSMA and RSMA); blue represents the left and right temporal cortex (LTC and RTC); yellow represents the left and right dorsolateral prefrontal cortex (LDLPFC and RDLPFC).

Figure 5 illustrates the results of functional connectivity during the WM tasks. The average functional connectivity in the AD group was significantly lower than that in the CN group (Figure 5E, *T* = 2.198, *p* < 0.05). Strong functional connectivity was observed in FP locations in both groups, as depicted in Figures 5C,D.

## 4 Discussion

This study aimed to elucidate the activation patterns of the frontotemporal cortex in the hemodynamic response to WM tasks using fNIRS in a sample of older adult patients with AD compared to CN subjects. We observed that in the two phases of the WM tasks, changes in HbO concentrations initially increased and then plateaued in the encoding and maintenance phases and finally increased to a much higher level in the retrieval phase in both AD and CN participants. These findings suggested that reduced cortical activation in the prefrontal and temporal cortex during the WM task might reflect the fact of cognitive decline in patients with AD. Moreover, in this study, we investigated cortical activations both in brain regions and in fNIRS channels, which might reflect more detailed physiological processes in relevant brain regions.

During the WM tasks, patients with AD exhibited lower cortical activation in both the encoding and retrieval phases compared to the CN group, as shown in Figure 3. Lower cortical activation during the encoding and maintenance phases is positively associated with poor memory function in patients with MCI and preclinical AD (Pavisic et al., 2021; Liu et al., 2023). However, during memory retrieval, neuronal activity peaked in both groups. Neuroimaging studies have also shown increased neural activity in the medial and lateral frontal cortices during memory retrieval (Cabeza et al., 2001). This heightened activity May be attributed to the involvement of more complex neurocognitive processes predominantly dependent on perceptual processes related to retrieval cues or tasks, as well as the recall of information from memory and related executive functions (Straube, 2012; Tabi et al., 2021).

Compared to the CN group, the average HbO concentration and functional connectivity were significantly lower in the AD group across different subregions or channels when performing the WM tasks. Brain network functions related to WM and attention decline even in the preclinical stages of AD (Lazarou et al., 2022). Moreover, patients with AD demonstrated decreased connectivity in specific networks during the memory retrieval phase (Li et al., 2021). However, an age-related increase in prefrontal cortex integration was associated with better retrieval performance in older adults, regarded as functional compensation (Deng et al., 2021).

A number of traditional non-invasive neuroimaging techniques, mainly including EEG, PET, and fMRI, have been used to assess resting-state functional connectivity (rsFC) in patients with AD (Allen et al., 2007; Zhao et al., 2022). Regarding the fNIRS-based rsFC, previous studies successfully validated the use of fNIRS in assessing rsFC in the human brain (Duan et al., 2012; Lu et al., 2010; White et al., 2009). In our study, rsFC was also assessed using fNIRS. Decreased rsFC was found in the FP area, the pars triangularis Broca’s area, the pre-motor and supplementary motor cortices, the temporal cortex, and the dorsolateral prefrontal cortex in the AD group compared to the CN group. The findings suggested that there is a difference in interhemispheric rsFC between AD and CN groups, which was consistent with previous studies (Mızrak et al., 2024). Regarding the powerful neuroimaging technique of EEG-fNIRS, whole-head EEG and frontal/prefrontal cortex fNIRS can also be used to evaluate brain activity in early AD and CN during WM tasks (Perpetuini and Chiarelli, 2020).

Recent studies have indicated sustained neuronal activity in the prefrontal, parietal, and occipital regions during WM tasks (Leavitt et al., 2017; Curtis and Sprague, 2021; van Kerkoerle and Self, 2017). In the present study, during the WM task performance, although the average HbO concentration in the left brain in the AD group was lower than that in the CN group, there was no significant difference between the two groups in the brain region scale. In channel scale, regarding the average HbO concentrations, compared to the AD group, cortical activations were higher in channels 7 and 32, which correspond to the frontal lobe in the CN group; regarding slope HbO concentrations, cortical activations in the CN group were higher in the channels 7, 8, and 9, which correspond to the prefrontal lobe, and channels 34, 38, and 23, which correspond to the frontal and temporal lobes. These findings suggested that fNIRS can provide more information on channel scales. These cortical activations during the encoding, maintenance, and retrieval phases of WM are positively associated with memory function (Cui et al., 2021; Uemura et al., 2016).

Multiscale biomarkers, including activities in brain regions or channels in this study, can provide more information to improve the identification of AD. Furthermore, multidimensional or multimodal diagnostic methods are potential hot research topics for future AD diagnosis (Li et al., 2023; Kim et al., 2022; Moallemian et al., 2023). Machine learning for cognitive outcome prediction from multimodal neuroimaging was also used to identify multimodal imaging biomarkers (Wang et al., 2023).

This study effectively examined the characteristic patterns of brain activity in patients with AD during the WM task using the fNIRS technique. Our findings provided evidence of cognitive impairment cognition and functional deficits in the prefrontal, local parietal, and temporal cortex in patients with AD. Additionally, our study suggests that fNIRS could serve as a reliable and valuable clinical diagnostic tool for AD. However, our study had certain limitations. First, the scope of the study was constrained by the limited number of channels. Thus, the area measured by fNIRS was restricted to the prefrontal and temporal cortices. Additionally, the small sample size limited the applicability of the findings. To address this, future research should use a larger sample size to enhance the generalizability of the results.

## 5 Conclusion

This fNIRS study demonstrated that patients with AD exhibited lower cortical activation in the prefrontal and temporal cortices and weaker functional connectivity during memory encoding and retrieval phases in the WM task compared to older adults in the CN group. These findings suggest that reduced cortical activation and neural connections detected by fNIRS might contribute to cognitive decline associated with AD. Additionally, this study suggests that fNIRS might be a potentially reliable method for diagnosing and screening AD.

## Data Availability

The raw data supporting the conclusions of this article will be made available by the authors, without undue reservation.
